# Th17/Treg Imbalance Induced by Dietary Salt Variation Indicates Inflammation of Target Organs in Humans

**DOI:** 10.1038/srep26767

**Published:** 2016-06-29

**Authors:** Tao Luo, Wen-jie Ji, Fei Yuan, Zhao-zeng Guo, Yun-xiao Li, Yan Dong, Yong-qiang Ma, Xin Zhou, Yu-ming Li

**Affiliations:** 1 Tianjin Key Laboratory of Cardiovascular Remodeling and Target Organ Injury, Pingjin Hospital Heart Center, Tianjin, P.R. China; 2 Department of Cardiology, No. 254 Hospital of PLA, Tianjin, P.R. China; 3 MRI Department, Pingjin Hospital, Tianjin, P.R. China

## Abstract

The functions of T helper 17 (Th17) and regulatory T (Treg) cells are tightly orchestrated through independent differentiation pathways that are involved in the secretion of pro- and anti-inflammatory cytokines induced by high-salt dietary. However, the role of imbalanced Th17/Treg ratio implicated in inflammation and target organ damage remains elusive. Here, by flow cytometry analysis, we demonstrated that switching to a high-salt diet resulted in decreased Th17 cells and reciprocally increased Treg cells, leading to a decreased Th17/Treg ratio. Meanwhile, Th17-related pathway was down-regulated after one day of high salt loading, with the increase in high salt loading as shown by microarray and RT-PCR. Subsequently, blood oxygen level-dependent magnetic resonance imaging (BOLD-MRI) observed hypoxia in the renal medulla (increased R2^*^ signal) during high-salt loading, which was regressed to its baseline level in a step-down fashion during low-salt feeding. The flow-mediated vasodilatation (FMD) of the branchial artery was significantly higher on the first day of high salt loading. Collectively, these observations indicate that a short-term increase in dietary salt intake could induce reciprocal switches in Th17/Treg ratio and related cytokines, which might be the underlying cellular mechanism of high-salt dietary induced end organ inflammation and potential atherosclerotic risk.

Salt is considered as an essential nutrient for humans. However, to our surprise, an increase in sodium has been identified as a dietary culprit for detrimental impacts on the vasculature, causing hypertension and impairing the functions of the heart and kidney^1^. Several recent studies have demonstrated that high levels of sodium could activate inflammatory innate and adaptive immune systems^2^,^3^. Thus, besides increasing blood pressure and the risk of stroke, high-salt dietary undermines the normal function of the immune response by promoting the development of T cells and inflammatory injuries to the target organs.

A third and fourth T cell subpopulation, designated T helper 17 (Th17) and regulatory T (Treg) cells, have emerged as independent differentiation pathways^2^,^4^,^5^. While the predominance of Th17 cells induces the secretion of a large number of pro-inflammatory cytokines, Treg cells restrict inflammatory responses and confer immune-tolerance^6^. The balance between Th17 and Treg cells depends on the activation of the transcription factors RORγt (factors retinoic acid receptor-related orphan receptor γt) and STAT3 (signal transducer and activator of transcription 3), or FoxP3 (forkhead box P3) and STAT5, respectively, which regulate the immune response through the secretion of pro- or anti-inflammatory cytokines^7^,^8^. Recently, it was demonstrated that high-salt intake could promote the development of IL-17-producing CD4^+^ T (Th17) cells, as an effector in response to extracellular bacterial infections and a critical mediator of autoimmune diseases^2^,^9^. In addition, regulatory T lymphocytes (Tregs) mediate peripheral tolerance and are involved in the maintenance of tolerance to self-antigens, which can be inhibited after high-salt dietary^10^. Collectively, an imbalance in Th17/Treg function induced by high salt intake is proposed to be implicated in inflammation and organ damages, however, very little is known about the involvement of these subpopulations in healthy subjects.

Serum/glucocorticoid regulated kinase 1 (SGK1) plays a key role in downstream activation of potassium, sodium and chloride channels and cellular stress response^11^. Transcriptional network analysis has identified SGK1 as a pivotal nodal point for the induction of Th17 cells, in tandem with contribution to hypertension and renal diseases^9^,^12^. SGK1 also has an effect on other T helper cell subsets^13^. Induction of pro-inflammatory Th17 cells by SGK1 enables the activation of p38/MAPK and NFAT5 pathways, which upregulate the expression of IL-23 receptor in a FOXO1-dependent manner but also strengthen the IL-17 inflammatory cascade^2^,^9^. Moreover, engagement of SGK1 activity, secondary to increased sodium chloride exposure, results in a Th1-type effector signature in Foxp3^+^ Tregs. This phenotype is marked by loss of suppressor function, whereas increased cellular proliferation and the secretion of cytokines^10^. Hence, excess NaCl might affect the adaptive immune system, especially causing the dysregulation of Th17/Treg ratio, via the SGK1 signaling.

Hypoxia is a common factor responsible for renal dysfunction and salt-sensitive hypertension^14^. It is estimated that more than 90% of renal oxygen consumption is used for tubular sodium transport via Na+/K+-ATPase^15^, which renders the kidney more susceptible to hypoxia in response to increased salt intake^14^. Hypoxia contributes to imbalance of Th17/Treg cells^16^ and exacerbates renal dysfunction. In addition, endothelial dysfunction, as an initial step in the development of atherosclerosis, has been associated with high salt intake^17^. High salt intake-enhanced production of vascular endothelia growth factor C (VEGF-C) has been demonstrated in animal models^18^. VEGF-A can promote accumulation of IL-17α-producing Th17 cell^19^. Moreover, dietary salt reduction has been shown to improve endothelial function assessed by flow-mediated dilatation (FMD)^20^. However, the immune-related mechanism underlying the correlation of salt intake with endothelial dysfunction is yet to be elucidated.

Therefore, the present work was designed to determine the relationship between: 1) variation in dietary salt intake and T cell subsets; 2) high dietary salt intake-induced changes in Th17/Treg-related cytokines and the inflammation of target organs. The endpoint was focused on functional dynamics of kidney and branchial artery, which were revealed by blood oxygen level dependent magnetic resonance imaging (BOLD-MRI) and flow-mediated vasodilatation (FMD), respectively. The findings might discover a pathophysiological network connecting dietary salt intake, adaptive immunity, and end organ inflammation.

## Methods

### Eligibility and Recruitment

A total of 15 healthy non-smoking male volunteers were randomly recruited by advertisement. Female subjects were not studied due to the impact of menstruation. The exclusion criteria included cardiovascular disease (stroke, heart failure, myocardial infarction and peripheral artery disease), diabetes mellitus, hematological disorders, cancer, current stage 2–3 primary hypertension (SBP ≥160 mmHg and/or DBP ≥100 mmHg), secondary hypertension, abnormal routine urinary test, previous or current abnormal renal function, and symptoms of upper respiratory tract infection and/or body temperature ≥37.5 degrees Celsius during investigation. All participants have provided written informed consent. The protocol was approved by the ethical committee of Pingjin Hospital, in accordance with the Declaration of Helsinki. All procedures were conducted according to the guidelines approved by the ethical committee at Pingjin Hospital.

### Study Protocol

**Figure 1:**
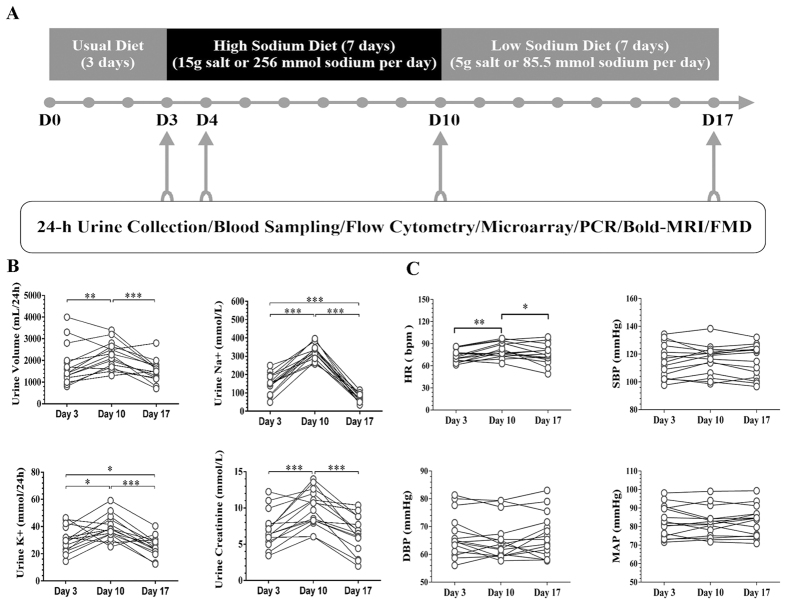
Research protocol and related clinical parameters. (**A**) Research protocol of the current study. (**B**) The time-dependent changes of 24 h urinary parameters. ^*^P < 0.05, ^**^P < 0.01, ^***^P < 0.001 (One way repeated measures ANOVA or Friedman test, n = 15). (**C**) Blood Pressure and Heart Rate Changes during Dietary Intervention (n = 15). SBP indicates systolic blood pressure; DBP indicates diastolic blood pressure; MAP indicates mean blood pressure; HR indicates heart rate. ^*^P < 0.05, ^**^P < 0.01 (One way repeated measures ANOVA or Friedman test, n = 15).

A three-phase dietary intervention study including usual-salt, high-salt and low-salt feeding was conducted. Our recent investigation in aged inhabitants in rural northern China demonstrated an average of 240 mmol Na^+^/day (~14 g salt) in 24 h urinary samples^21^. Considering sodium loss during sweating, 15 g salt (256 mmol Na^+^) per day was defined as the high-salt loading. The low salt level was based on World Health Organization’s recommendations (5 g salt or 85.5 mmol Na^+^/day). During the first 3 days, participants were required to eat all their meals in the hospital’s cafeteria. From day 4 to day 17, all foods were prepared by professional dietitians and provided by the investigators. The participants were required to eat their meals at a defined place while being monitored by the investigators. A detailed research protocol was described in Fig. 1A.

### Blood Pressure Measurement and 24 h Urine Collection

Blood pressure and heart rate were measured by the same investigator between 8:00–10:00 am and recorded by an automatic Omron HEM-7200 device (Omron Inc., Dalian, China), where the subject was in a sitting position after 10 min of rest. Participants were asked to provide six samples of 24 h urine in a 4000 mL wide-neck plastic container (days 3, 4, 10, 11 and 17), to examine urinary volume as well as the concentration of sodium, potassium and creatinine.

### Flow cytometry analysis of B cell and T cell Subsets

Flow cytometry analysis of circulating B and T cell subsets from the 15 participants was performed according to a modified version of previously published protocols based on CD19 and CD4 gating strategies^22^. Briefly, a 21-G needle and a light tourniquet were used for blood sampling via the antecubital vein, and the first 10 mL of blood was used for biochemical assays and PBMCs isolation using the Ficoll gradient method (HistopaqueH-1077, Sigma-Aldrich, St Louis, MO, USA). Blood for flow cytometry analysis was gently transferred to sodium citrate anticoagulated tubes. Th17/Treg cell phenotype assay was shown as an example: 50 μL whole blood was incubated with an antibody mixture containing FITC labeled anti-human FoxP3 (clone 206D), PE-labeled anti-human IL-17 (clone BL168), Cy5-labeled anti-human CD4 (clone OKT4) and Cy7 labeled anti-human CD25 (clone BC96) for 15 min at room temperature, and kept protected from light. Then, 1 mL red blood cell lysis buffer was added and the solution was incubated for 10 min. The following isotype controls were used: mIgG1-FITC (clone MOPC-21), mIgG2a-PE (clone MOPC-21), mIgG2b-PC5 (clone MPC-11), and mIgG1-PC7 (clone MOPC-21). All antibodies were purchased from BioLegend (San Diego, CA, USA).

**Figure 2:**
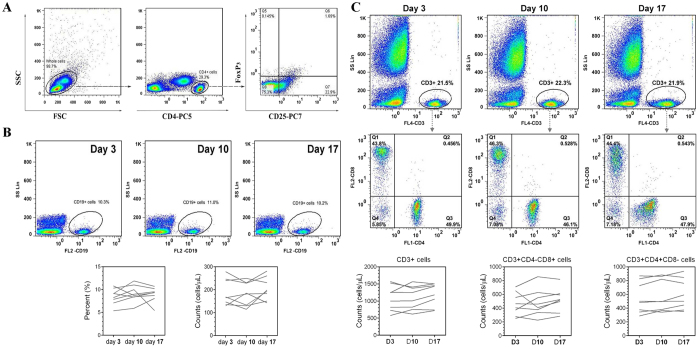
Flow cytometric detection of B and T cell subsets. (**A**) Gating strategies for Treg lymphocyte subset analysis as an example to illustrate the methods of gating strategies for B and T subsets analysis. Left plot shows the SSC/FSC plot for whole cells after red blood cell lysis. Middle plot shows the gating of CD4^+^ cells. Right plot shows Treg population was backgated into the CD4^+^ plot. (**B**) B cell subsets analysis. No changes during dietary intervention. (**C**) T cell subsets (CD3, CD4 and CD8) analysis. No changes during dietary intervention.

Unstained, single stained and Fluorescence Minus One (FMO) controls were used for setting compensation and gating boundaries. 50 mL Flow-Count^TM^ fluorescent microbeads (Beckman-Coulter, Miami, FL, USA) were added for absolute counting. Data were acquired using a Cytomics FC500 cytometer (Beckman-Coulter, Miami, FL, USA) and analyzed using FlowJo software (Treestar, Ashland, OR, USA). At least 150, 000 events were collected in each sample. Gating strategy for Treg lymphocyte subset analysis was shown in Fig. 2A as an example to illustrate the methods of gating strategies for B and T cell subsets analyses. The laboratory coefficient of variation was 2.0% for absolute B and T count by flow cytometry, and <5.0% for surface markers.

### Gene Expression Profiling and Validation of Differential Genes Expression

Blood samples collected were used for total RNA extraction and gene expression profiling analysis. Total RNA of PBMCs was isolated using Trizol (Invitrogen, Carlsbad, CA, USA) according to the manufacturer’s protocol. The quality and concentration of the RNA were determined using an RNA Nano Chip Kit and a 2100 Bioanalyzer (Agilent Technologies, Santa Clara, CA, USA). RNA purity (ratio of absorbance at 260 nm and 280 nm) was accessed using the NanoVue spectrophotometer (GE Healthcare Life Sciences). Total RNA was then reverse-transcribed by incubating with Oligo-(dT) primers (Roche, Basel, Switzerland) and M-MLV reverse transcriptase (Promega, Madison, WI, USA) according to the manufacturer’s instructions.

Microarray analysis was performed using Agilent Whole Human Genome Microarray 4 × 44 K arrays (design ID 014850). The gene list, annotation and probe sequences are described in eArray application at http://www.genomics.agilent.com. Labeling of probes with cyanine 3 dye (Cy3), hybridization and washing procedures followed strictly the manufacturer’s protocol (One Color Quick Amp Labeling Kit, Agilent Technologies). The arrays were scanned using a GenePix 4000B Microarray Scanner (Molecular Devices) using default parameters for Agilent 44K microarrays. Initial data analysis was performed using the Agilent Feature Extraction software (v9.5). The quality of the microarray data was assessed using the positive controls and RNA spike-ins. The gProcessedSignal (i.e. end result of standard Agilent Feature Extraction normalization and background correction procedures) from each array was loaded into the Partek Genomics Suite (v6.6), adjusted between arrays using quantile normalization, and log transformed for further analyses. Principal Components Analysis (PCA) was used as an exploratory tool to identify major effects.

To identify differentially expressed genes of interest, real-time PCR (RT-PCR) was used to validate the microarray results. RT-PCR reaction was performed in triplicate using PowerH SYBR green Master Mix (Roche, Switzerland) on an ABI Prism 7300 system (Applied Biosystems, Foster City, CA, USA). All values were normalized to the housekeeping gene glyceraldehyde phosphate dehydrogenase (GAPDH) and calculated with the 2^−ΔΔCT^ method^23^.

### Renal BOLD-MRI

A total of 15 participants finished serial MRI on days 3, 4, 10 and 17, using a Phillips Intera 3.0 Tesla whole body magnetic resonance system (Philips Medical Systems, Netherlands) with a 6-channel flexible matrix coil to receive the MR signal, according to previously described methods^24^. Coronal and axial T2^*^-weighted images were acquired in order to cover two kidneys. BOLD-MRI T2^*^-weighted images were recorded during a single breath holding with a respiratory-triggered Fast Field Echo (FFE) sequence. BOLD-MRI images were analyzed based on a modified protocol from previous publication^25^. Eight images were selected in which the cortex and medulla anatomic boundaries were clear from 20 T2^*^ weighted images; and the R2^*^ maps were calculated using ImageJ (NIH, Bethesda, MD, USA) on a pixel-by-pixel basis by fitting the corresponding echo time. Regions of interest (ROI) with unfixed size (60–90 pixels) were defined at the upper, middle and lower poles of both kidneys in the medulla and cortex based on the anatomical images. ROIs in the medulla and cortex were selected and measured independently by 2 experienced investigators.

### Flow-mediated Vasodilatation (FMD) of The Brachial Artery

FMD of the branchial artery was performed according to a modified protocol^26^. Briefly, the right shoulder and arm of the subjects were positioned on soft supports for optimal comfort and stability, to avoid muscle tension build-up and a subsequent movement. Upper arm and wrist were rested on soft supports to avoid positioning artifacts due to cuff-inflation. A well-accessible segment of the right brachial artery from the cubital fossa at 5–15 cm was interrogated using a 7.5 MHz, 40 mm lineararray ultrasound system (GE Vivid E9, GE HealthCare, USA) in Duplex-mode to record simultaneously artery diameter and center-line blood flow sonogram. End diastolic diameter (D_0_) of the branchial artery was measured at the ECG R wave apex. The medical blood pressure meter cuff was positioned on the right lower arm and inflated to 280 ~ 300 mmHg for 5 minutes to elicit upon release the hyperemic response for 60 ~ 90s required for continued brachial artery flow-mediated dilation measurement of end diastolic diameter (D_1_). During inspection process, measuring probe was fixed on the same position, and instrument parameters were unchanged. Three cardiac cycles were measured and an average value taken to evaluate endothelium-dependent diastolic function. FMD = (D_1_ − D_0_)/D_0_ × 100%.

### Statistical Analysis

The Shapiro-Wilk test was used to assess normality of quantitative variables. Continuous variables with normal distributions were presented as the mean ± SD or median with interquartile range. For microarray assay, ANOVA implementation of Partek was used. ANOVA model was defined by the experimental design and included variations of sample collections (D3, D4). Since RNA extraction was not performed in the same day, ANOVA was used to address the bias caused by batch effects. The raw microarray data (background-corrected signal) can be assessed at Gene Expression Omnibus (GEO accession GSE48080). To test the differences across time, a one-way repeated-measures ANOVA with Newman-Keuls post hoc analysis was used. If the data failed in normality tests, a Friedman test followed by a Dunn’s test for multiple comparisons was performed. Correlations between two continuous variables were calculated using Pearson’s or Spearman’s correlation coefficient. All statistical analyses were performed using GraphPad Prism version 5 (GraphPad Prism Software Inc., San Diego, CA, USA). A two-tailed *P* value of <0.05 was considered statistically significant.

## Results

### Biological and Physiological Changes during Dietary Intervention

A total of 15 volunteers were enrolled, who provided 24 h urinary and blood samples at baseline. All subjects agreed to participate in and finished the dietary intervention study. The characteristics of the population were as follows: 15 men, mean age of 26.93 ± 0.92 years, mean body mass index (BMI) of 22.9 ± 0.68 kg/m^2^ and mean waist-to-hip ratio of 0.84 ± 0.02. The dynamic changes in parameters of 24 h urinary samples were summarized in Fig. 1B. The salt intake at baseline, high-salt phase and low-salt phase, as determined by 24-h urinary sodium, was significantly different. There was no obvious change in blood pressure, whereas heart rate was significantly increased during the high-salt period and regressed to baseline level during the low-salt intervention (Fig. 1C). Serum/plasma biochemical alterations as follows (data not shown): In general, lipid profiles remained stable, despite a slight decrease of high density lipoprotein (HDL) cholesterol. There was a statistically suppressed serum aldosterone level during the high-salt period, while the dynamics of plasma renin activity and angiotensin II did not reach statistical significance.

### B and T cell Subsets Analyses

**Figure 3:**
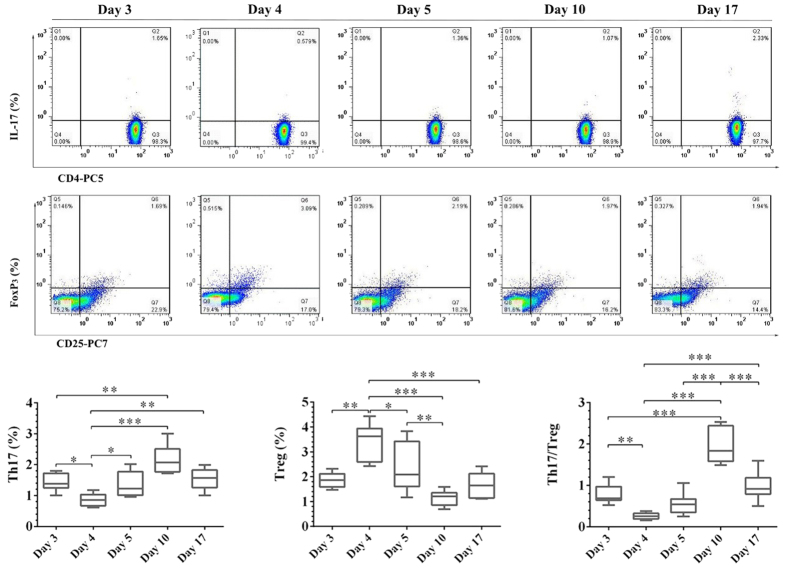
Representative flow cytometry analysis of Th17 and Treg and related statistical comparisons. The box-and-whisker plots: the boxes extend from the 25th to the 75th percentile, with a line at the median. The whiskers extend above and below the box to show the 5th–95th percentiles of values. ^*^P < 0.05, ^**^P < 0.01, ^***^P < 0.001 (one way repeated measures ANOVA or Friedman test, n = 15).

Dynamics of B cell subsets were shown in Fig. 2B. With regard to total B cell count and percentage, no significant change was observed during the dietary salt intervention. Similar patterns were observed in CD3^+^, CD4^+^ and CD8^+^ T cell subsets as shown in Fig. 2C. However, Th17 and Treg showed unique dynamics, undergoing a progressive change during high-salt feeding before abruptly decreasing up to day 17. Specifically, Th17 counts were decreased by net increase in dietary salt intake between day 3 and day 4, and then gradually increased during the high salt period. Towards opposite direction, Treg counts were increased on day 4 and then decreased throughout the whole high salt period. Interestingly, Th17/Treg ratio was significantly increased from day 4 to day 10, in spite of decreasing gradually to the baseline on day 17 (Fig. 3).

### Candidate Genes Identification by Microarray Analysis

**Figure 4:**
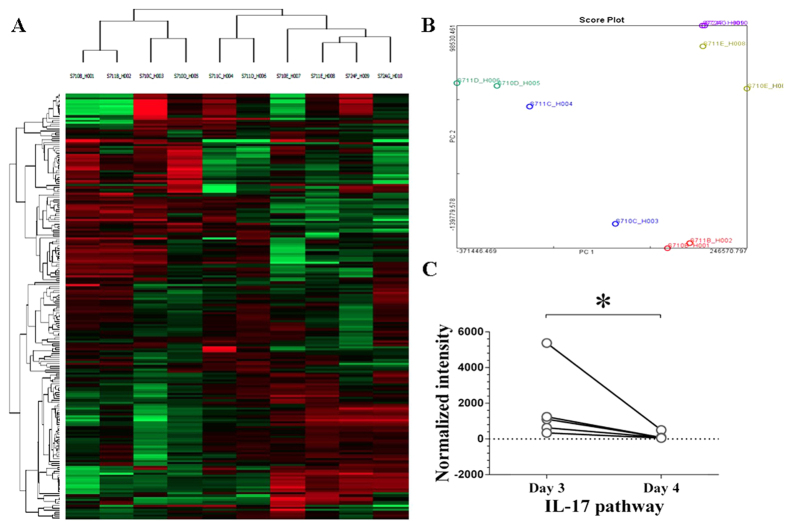
Gene expression profiling. (**A**) Clustering analysis. Clustering was performed to visualize the correlations among the replicates and varying sample conditions. Up- and down-regulated genes are represented in red and green colors, respectively. (**B**) PCA plot. The variable of the first three principal components (PC1, PC2, PC3) for this study are 71.41%, 13.47%, and 5.90%, respectively. (**C**) Downregulated IL-17 pathway. ^*^P < 0.05.

Blood samples form 5 subjects that met the sampling criteria were included in the microarray. Principle Component Analysis (PCA) was performed to evaluate the differences among biological replicates and treatment conditions (Fig. 4B). For advanced data analysis, all biological replicates were pooled and calculated to identify differentially expressed genes based on the thresholds of fold changes and p-values. The correlation of expression profiles between biological replicates and treatment conditions was demonstrated by unsupervised hierarchical clustering analysis (Fig. 4A). A subset of differential genes was selected for clustering analysis. An intensity filter was used to select genes where the difference between the maximum and minimum intensity values exceeds 2500 among all microarrays. Hierarchal clustering analysis revealed that the differences could be successfully distinguished between day 3 and day 4 groups (Fig. 4A). Microarray analyses identified a total of 210 transcripts differentially expressed in day 4 group compared to day 3 group. Gene ontology (GO) hierarchy analysis revealed significant functions involved in T cell activation signaling pathway (p = 0.002, enrichment = 134.58), T cell differentiation (p = 0.003, enrichment = 107.39) and regulation of T cell activation signaling pathway (p = 0.027, enrichment = −67.61). Among these GO categories, 4 differentially expressed salt-sensitive genes were identified: CD247, nuclear factor of activated T-cells 5 (NFAT5), vascular endothelial growth factor (VEGF) and serum/glucocorticoid regulated kinase 1 (SGK-1). Additionally, gene set enrichment analysis was performed according to previous selected differentially expressed gene lists identified from the KEGG database (*p* < 0.05), including the CTL (p = 0.0003, enrichment = 101.38), IL17 (p = 0.0008, enrichment = 125.76) and CTLA4 pathways (p = 0.012, enrichment = 68.34). Totally 5 differentially expressed IL-17/Treg genes were found: IL-23, IL-8, T-cell activation GTPase activating protein (TAGAP), CD247 and NF-κB (Fig. 4C). These genes were selected as the candidate genes for the following experiment. Pathway and gene ontology analysis was shown in Table 1.

### Verification of candidate genes by RT-PCR

**Figure 5:**
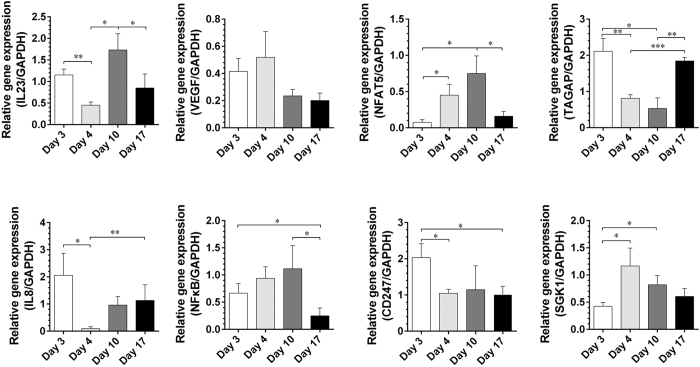
Activation of candidate genes during dietary intervention: related gene expression by real-time PCR (n = 15, one way repeated measures ANOVA). Day 3 to Day 17 were labeled in the x-axis. ^*^P < 0.05, ^**^P < 0.01, ^***^P < 0.001. NF-κB, nuclear factor kappa B; TAGAP, T-cell activation GTPase activating protein; SGK1, Serum/Glucocorticoid Regulated Kinase 1; CD247, CD247 molecule; IL-8, Interleukin 8; IL-23, Interleukin 23; VEGF, vascular endothelial growth factor; NFAT5, Nuclear factor of activated T-cells 5; GAPDH, glyceraldehyde-3-phosphate dehydrogenase.

Candidate genes expression profiling was similar to the microarray analysis. As shown in Fig. 5, mRNA levels of NFAT5 and SGK-1, as high-salt sensitive genes, were up-regulated during the high-salt phase, which could be reversed by a low-salt diet. Additionally, VEGF, a stimulator of vasculogenesis and angiogenesis, was up-regulated on day 4 (one day after high salt loading). Similarly, inflammatory gene NF-κB showed an upward trend on day 4, statistically up-regulated on day 10, but down-regulated on day 17. Interestingly, IL-23, TAGAP, IL-8 and CD247, involved in IL-17 pathway, were all significantly down-regulated on day 4, and returned to the level of day 3 on day 17, except CD247.

### BOLD-MRI for Renal Hypoxia

**Figure 6:**
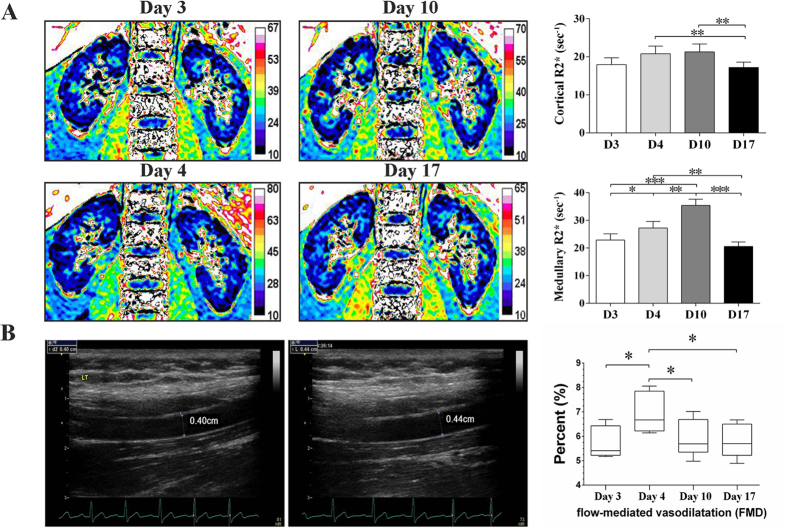
Renal blood oxygen level dependent-magnetic resonance imaging (BOLD-MRI) and brachial artery reactivity during dietary intervention. (**A**) BLOD-MRI during dietary intervention. The representative changes of BOLD-MRI images from one participant during dietary intervention on day 3, day 4, day 10 and day 17, respectively. R2^*^ signal changes in renal cortex and medulla, respectively. Statistical comparisons were derived from 15 subjects by one way repeated measures ANOVA. ^*^P < 0.05, ^**^P < 0.01, ^***^P < 0.001. D3 to D17 labeled in the x-axis indicated day 3 to day 17. (**B**) Brachial artery reactivity during dietary intervention. Left panel: Diameter of brachial artery before releasing the hyperemic response. Diameter of brachial artery after releasing the hyperemic response. Mid-panel: Dynamics of FMD from day 3 to day 17. Right panel: The box-and-whisker plots: the boxes extend from the 25th to the 75th percentile, with a line at the median. The whiskers extend above and below the box to show the 5th–95th percentiles of values. ^*^P < 0.05 (one way repeated measures ANOVA or Friedman test, n = 15).

Figure 6A demonstrated a remarkable and progressive increase in renal medullary R2^*^ signals (decreased tissue oxygenation) during high-salt loading. Notably, the increased renal medullary R2^*^ signal regressed to its baseline level in a step-down fashion during low-salt feeding. The renal cortical R2^*^ signal remained unchanged during the high-salt phase, whereas at the end of low salt phase, the cortical R2^*^ signal was significantly reduced compared with the high-salt period.

### FMD for Brachial Artery Vascular Reactivity

As shown in Fig. 6B, the diameter of brachial artery measured on day 4 was remarkably increased compared with that on day 3. The calculated FMD value was statistically significant. However, the increased FMD value regressed to its baseline level at the low salt phase. Notably, FMD value at the end of high salt phase was reduced compared with that on day 4 (*P* < 0.05).

## Discussion

Reciprocal switch of Th17/Treg cells has been indicated to play a pivotal role in adaptive immune function. Here, for the first time, our study demonstrates that abrupt switch to a high-salt diet from normal diet resulted in decreased Th17 cells and a reciprocal increase in Tregs count, leading to a decreased Th17/Treg ratio by flow cytometry analysis. Meanwhile, Th17-related pathway was down-regulated after one day of high salt loading, with the increase in high salt loading by using microarray analysis and RT-PCR. Subsequently, blood oxygen level-dependent magnetic resonance imaging (BOLD-MRI) observed hypoxia in the renal medulla (increased R2^*^ signal) during high-salt loading, which was regressed to its baseline level in a step-down fashion during low-salt feeding. The Flow-mediated vasodilatation (FMD) of the branchial artery was significantly higher on the first day of high salt loading. As anticipated, these alterations could be quickly reversed when transitioned to a low-salt diet, whereas the blood pressure remained unchanged during dietary intervention.

As a matter of fact, the plasma sodium concentration is normally maintained within a narrow physiological range by rigorous control systems. However, emerging evidence suggests that a transient elevation in plasma sodium concentration and plasma osmolality would occur when high salt diet is consumed^27^. To determine the effect of NaCl concentration elevation on adaptive immune system, blood samples from subjects was adopted for flow cytometry analysis. We found that B cell count and percentage, as well as CD3^+^, CD4^+^ and CD8^+^ T cells, had no significant change during the salt intervention study. This implied that short-term salt intervention might not affect immune system. Of note, subpopulation of T cells, Th17/Treg ratio was significantly changed when abruptly switching from low-salt to high salt-diet. To some extent, we had some different results of flow cytometric detection compared with other studies^28^,^29^. According to Gavin MA *et al*. in PNAS, 2006^28^, Treg is inferred to account for about 1% to 3% of CD4 cells, which is consistent with our results. Moreover, different antibodies against mouse and rabbit species were used in this study. Additionally, Gavin MA *et al*. in PNAS, 2006^28^ also mentioned that using the rabbit antibodies raised the percentage of Treg up to 6%. Considering the use of human antibodies in our study may generate different results. In Gavin MA *et al*. in PNAS, 2006^28^, the shift of CD25 positive cells results from comparison between normal and Foxp3 defect population. Their data demonstrated that Foxp3 expression was increased only in CD25^high^ CD4 cells, while in CD25^low^ CD4 cells, Foxp3 expression was rare. Murine models for Th17 staining from Dong C’s lab^29^ may generate different results from our study conducted in human. Recent studies have indicated that Tregs might suppress proliferation and cytokine production by regulating target cytotoxic T cells^30^,^31^ and antigen presenting cells (APCs)^32^. Treg cell suppression can be achieved in both cell-contact-independent (e.g., via production of inhibitory cytokines and deprivation of IL-2 and ATP/ADP) and cell-contact-dependent manners (e.g., induction of cytolysis and modulation of APCs)^33^. In the present study, we observed that reciprocal relationship between Treg and Th17 cell altered significantly with abrupt high-salt challenge, suggesting that short-term NaCl loading may lead to an imbalance between Th17 and Treg cells. Interestingly, Tregs increased along with the decrease of Th17 after one day high salt loading, indicating that Tregs induced immune tolerance quickly by responding to inflammatory cues^34^, as a potential mechanism underlying their contribution to poor expansion of Th17 during high salt intake.

From microarray analysis, we identified 210 transcripts differentially expressed after one day high salt loading. Subsequently, these candidate genes were validated by RT-PCR, in accordance with flow cytometry analysis. SGK1, acts as a mediator for sodium homeostasis^35^,^36^, was upregulated during the high salt phase, in agreement with that SGK1 activation promoted Th17 cell polarization in an EAE model^2^,^9^. Hypertonic salt directly activates the kinase SGK1, which stabilizes IL-23R and thus reinforces the Th17 phenotype. In an experimentally induced asthma murine model, SGK1 activation favored Th2 phenotype polarization, and SGK1 deletion in T cells was protective against asthma^13^. The Rel-like transcription factors NF-κB and the calcineurin-dependent NFATc proteins regulate thymocyte development downstream of the pre-TCR^37^. Thymocytes also express the calcineurin-independent NFAT protein NFAT5, which has hybrid features of both NF-κB and NFATc proteins^38^. NFAT5 protects cells from osmotic stress^39^, and NFAT5-deficient mice present severe atrophy of the renal medulla, systemic hypernatremia, and a reduced thymocyte compartment and mature T-cell lymphopenia^40^. With a high salt diet, NF-κB and NFAT5 were up-regulated at the mRNA level in the present study. It is known that hypertonic stress in mammals is sensed through p38/MAPK, the ancient yeast hypertonic stress response element^41^. The key translator of this cascade is the osmo-sensitive transcription factor NFAT5^42^. Our microarray analysis indicated the stimulation of both inflammatory and classic hypertonicity induced pathways. The CD4^+^ cells expressed high levels of the NFAT5 target gene SGK1^43^. SGK1 expression was strongly induced by high salt on the first day following stimulation of naive T cells under Th17-polarizing conditions. This was followed by a moderate decline at the end of high salt diet to a steady level substantially higher than the baseline. Furthermore, SGK1 was specifically induced and maintained by exposure to IL-23, gradually increasing during the high salt phase. Thus, IL-23 signaling may be critical for maintaining SGK1 expression during Th17 cell differentiation.

We have identified that TAGAP was decreased throughout the high salt phase. TAGAP is expressed on activated T-cells, however, its role in T-cell biology remains unclear^44^. TAGAP SNPs were associated with the susceptibility to immune mediated disease^45^. The ζ-chain of T cell receptor (CD247), a part of T-cell receptor-CD3 complex on T cells and activating receptor on NK cells^46^, as well as IL-8 (stimulated by IL-17 on human fibroblasts) were both down-regulated after one day high salt loading. These candidate genes may involve in Th17 pathway. In addition, VEGF had an upward trend at the beginning of high salt diet, and regressed to the baseline level at the end. Our results support the notion that extracellular hyperosmolarity stimulates VEGF gene transcription and the secretion of VEGF protein, dependent on the activation of NFAT5^47^.

To determine the association between the status of T cell subpopulation and end organ inflammation, we examined renal alterations by BOLD-MRI, a non-invasive and reliable approach for the assessment of renal hypoxia. It utilizes the magnetic properties of hemoglobin when the oxygenated converts to deoxygenated form^48^. Consistent with previous work^49^ (where the salt loading and depletion were separated with a wash-out period), dietary salt intake influenced renal oxygenation. Moreover, switching from normal salt to high-salt (~9 g/day to ~18 g/day) and transition from high-salt to low-salt (~5 g/day) could change the renal medullary R2^*^ values (inversely correlated with renal tissue oxygenation level). BOLD-MRI signal is the presentation of “net” tissue oxygen bioavailability, which cannot differentiate between changes in oxygen delivery, consumption, utilization efficiency and diffusion^50^. In this regard, the initial increase in R2^*^ signal after one day of high-salt loading might be the consequence of increased oxygen consumption, which maintained renal hypoxia during high-salt loading. Further increases in the R2^*^ signal might be due to oxidative stress (since sodium intake remained constant during high-salt loading), which reduced mitochondrial oxygen utilization and nitric oxide bioavailability, leading to renal hypoxi^51^. A recent study has demonstrated that VEGF decreased after renal ischemic insult^50^, suggesting that renal hypoxia is closely associated with high-salt loading. Importantly, urinary CD247^46^ was downregulated in peripheral lymphocytes from patients with long-term surviving kidneys, presenting similar changes to our study in renal oxygenation and circulating T cell subpopulation.

In the present study, we also demonstrated a parallel relationship between FMD changes and VEGF dynamics in response to a variation in dietary salt intake. The FMD value in response to the first day high-salt loading was significantly increased, and regressed to baseline level at the end of high-salt loading. Salt intake and FMD value didn’t obviously affect the blood pressure. We speculate that the salt may acutely affect endothelial functions via an alteration in plasma sodium. Increasing plasma sodium concentration within a narrow physiologic range (132–145 mmol/L) was shown to stiffen human endothelial cells and reduce nitric oxide (NO) production^52^. NO is critical for downstream signaling of VEGF-induced endothelium-dependent diastolic function^53^. In conduit arteries, FMD is principally mediated by endothelium derived NO^54^. Notably, VEGF was increased after one day high-salt loading, and then abruptly decreased at the end of the high-salt phase, similar to that of the low-salt phase. This phenomenon highlights the unique feature of VEGF in sharp salt fluctuation. However, a causal role of high salt in endothelium dysfunction needs to be explored.

There is no direct evidence that Th17/Treg imbalance could cause microscopic or macroscopic injuries to kidneys and vascular endothelium, as a limitation of the present study. Our findings indicated that inflammatory factors might implicate renal or vascular endothelial damage through Th17/Treg pathways. Pathological changes in kidney mediated by high salt diet may occur in a long intervention period, however, in our present study for a short period, related target organ injuries might be attributed to inflammatory and immune disorder in blood circulation. Based on our study, influences on kidneys by disturbances in metabolic homeostasis were required to be investigated. Our future plan includes *in vivo* animal models to clarify candidate molecular signaling pathways related to high-salt dietary.

**Figure 7:**
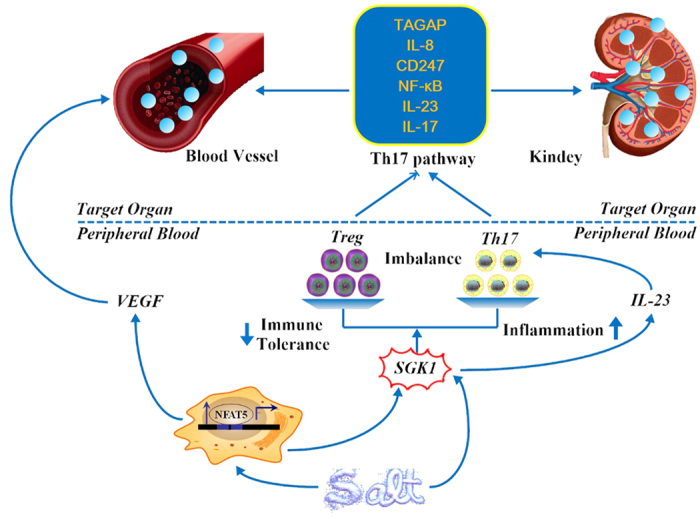
Hypothesized impact of high dietary sodium on immune system and target organs. Hypertonic saline has a double effect on reciprocal switches of Th17/Treg: it directly induces the kinase SGK1, which reinforces the Th17 phenotype by IL-23, and it also activated nuclear factor of activated T cells 5 (NFAT5) transcription factor, which induce expression of SGK1. By means of the enhanced production of cytokines, such as interleukin-23, interleukin-8, interleukin-17, CD247, NF-κB and T-cell activation GTPase activating protein (TAGAP), the stimulated Th17-related pathway may modulate the host defense and exacerbate target organ injuries. In addition, high-salt dietary may exert detrimental impacts on vasculature by modulating the secretion of vascular endothelial growth factor (VEGF) via NFAT5 activation.

Taken together, we have identified reciprocal switches of Th17 and Treg cells, as well as the activation of pro-inflammatory cytokines in response to increased dietary salt intake. In addition, T cell subpopulation, especially the ratio of Th17/Treg, is associated with high-salt intake induced renal hypoxia and vascular endothelium dysfunction (related mechanisms shown in Fig. 7). A temporal and spatial correlation was clearly demonstrated in a small sample size, whereas a causal linkage between Th17/Treg and renal function in response to fluctuation in dietary salt should be verified in a large population. Our findings may reveal a novel pathophysiological network involving dietary salt intake, innate immunity and target organ inflammation.

## Additional Information

**How to cite this article**: Luo, T. *et al*. Th17/Treg Imbalance Induced by Dietary Salt Variation Indicates Inflammation of Target Organs in Humans. *Sci. Rep.* **6**, 26767; doi: 10.1038/srep26767 (2016).

## Figures and Tables

**Table 1:** Pathway and Gene Ontology Analysis.

Geneset Name	Genes inOverlap (k)	P value
KEGG_T_CELL_RECEPTOR_SIGNALING_PATHWAY	17	0.000007^***^
BIOCARTA_CTL_PATHWAY	4	0.0003^***^
BIOCARTA_IL-17_PATHWAY	5	0.0008^***^
T_CELL_ACTIVATION	7	0.002^**^
T_CELL_DIFFERENTIATION	4	0.003^**^
BIOCARTA_CTLA4_PATHWAY	3	0.012^*^
LYMPHOCYTE_ACTIVATION	7	0.013^*^
REGULATION_OF_LYMPHOCYTE_ACTIVATION	5	0.014^*^
LYMPHOCYTE_DIFFERENTIATION	4	0.021^*^
REGULATION_OF_T_CELL_ACTIVATION	4	0.027^*^

Top 10 enrichment pathway and GO terms for gene set enrichment analysis and GO analysis, respectively. ^*^P < 0.05, ^**^P < 0.01, ^***^P < 0.001 (One way repeated measures ANOVA or Friedman test).
